# Adenocarcinoma arising in a cystic duplication of the small bowel: case report and review of literature

**DOI:** 10.1186/1477-7819-10-55

**Published:** 2012-04-10

**Authors:** Gregor Blank, Alfred Königsrainer, Bence Sipos, Ruth Ladurner

**Affiliations:** 1 Department of General, Visceral and Transplant Surgery, University of Tübingen, Hoppe-Seyler-Straße 3, D-72076 Tübingen, Germany; 2 Institute of Pathology, University of Tübingen, Liebermeisterstraße 8, D-72076 Tübingen, Germany

**Keywords:** Enteric duplication, Duplication cyst, Malignant change, Small bowel cancer, Adenocarcinoma

## Abstract

Enteric duplications are rare, but can occur anywhere along the digestive tract. Most of the patients become symptomatic in early childhood and only a few cases of adult patients have been reported in literature. Here we report a unique case of an adenocarcinoma arising in a coincidentally found cystic duplication of the small bowel.

## Background

The term 'alimentary tract duplication' was characterized by W.E. Ladd to describe those congenital malformations that involve the mesenteric side of the associated alimentary tract and share a common blood supply with the native bowel [1]. Enteric duplications are unusual, but can occur anywhere along the digestive tract [2-7], most frequently found around the ileocoecal region [2-7]. Most patients become symptomatic within the first year of life [2-5]. Reports of enteric duplications in adulthood are extremely scarce in English language literature [8]. Although rare, malignant change can occur within the intestinal duplication [9]. In this report we present a case of an adenocarcinoma arising in a coincidentally found cystic duplication of the small bowel.

## Case presentation

**Figure 1:**
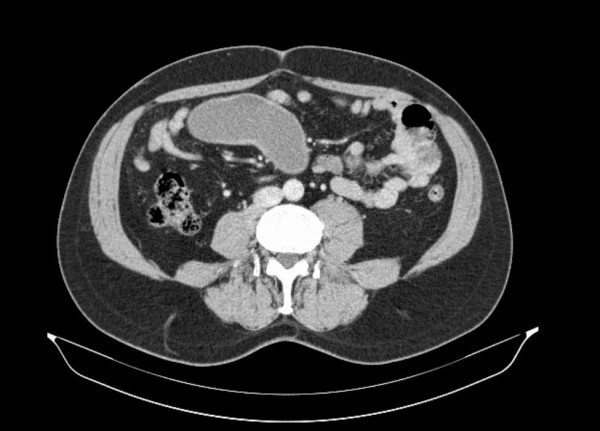
**Computed tomography scan showing a low-density cystic mass (4 × 10 cm) with an enhanced margin in the mid-abdomen**.

A 51-year-old man with no abdominal symptoms was admitted to our hospital with an external computed tomography (CT) scan showing a cystic mass in the mid-abdomen (Figure 1). The cystic mass was low-density and had enhanced margins. The size of the structure was measured at 4 × 10 cm and it was located in the ileal mesenterium. The differential diagnosis contained a mesenteric cyst, a Meckel's diverticulum, and an enteric duplication.

Physical examination was unremarkable but laboratory tumor marker levels were slightly elevated: carcinoembryonic antigen (CEA) 13.2 μg/l (standard value < 5 μg/l) and CA19-9 55 kU/l (standard value < 37 kU/l).

**Figure 2:**
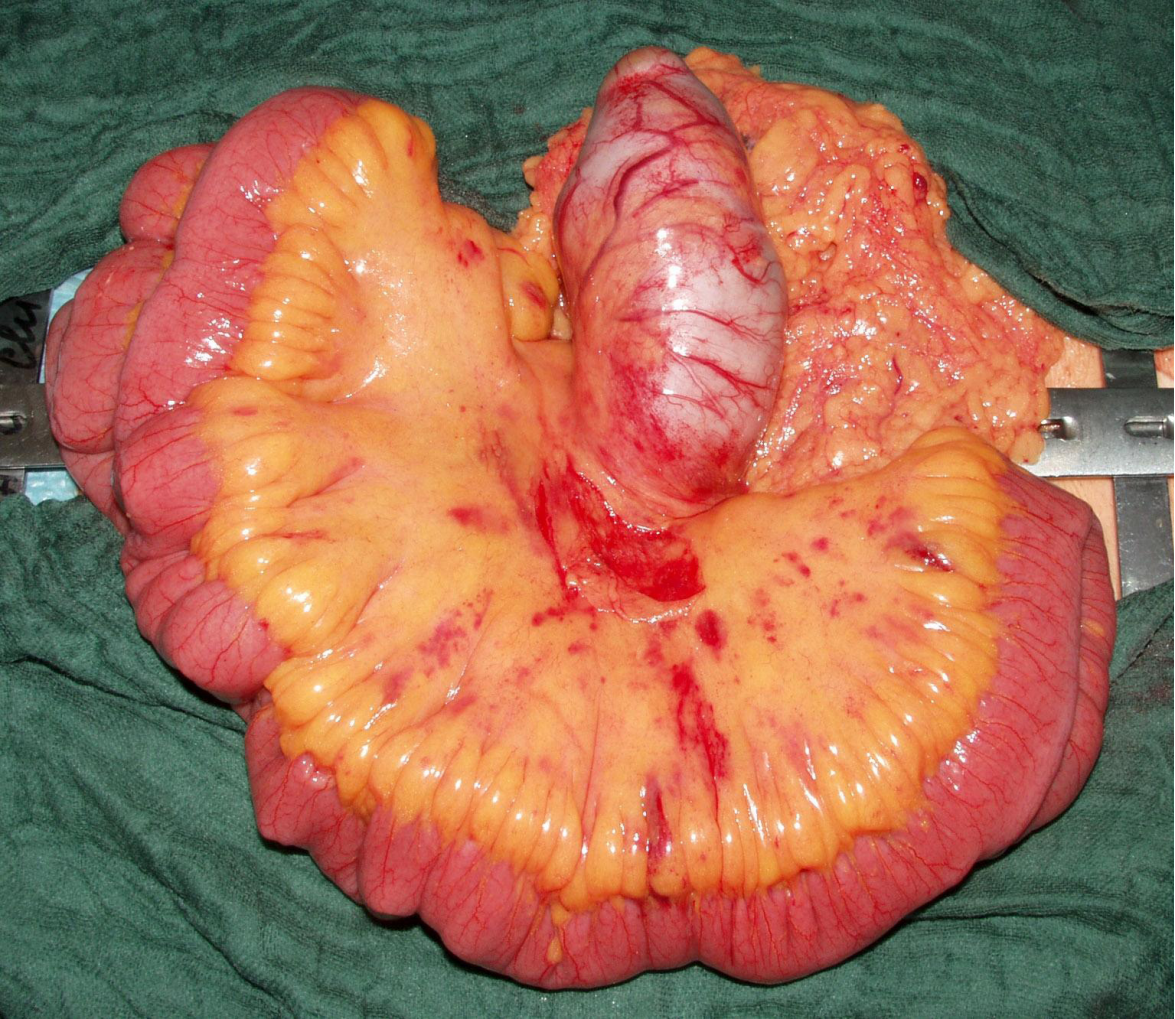
**Intraoperative photograph showing the cystic mass in the mesenterium without a connection to the surrounding small bowel**. The cystic mass bears great similarity to the small bowel.

**Figure 3:**
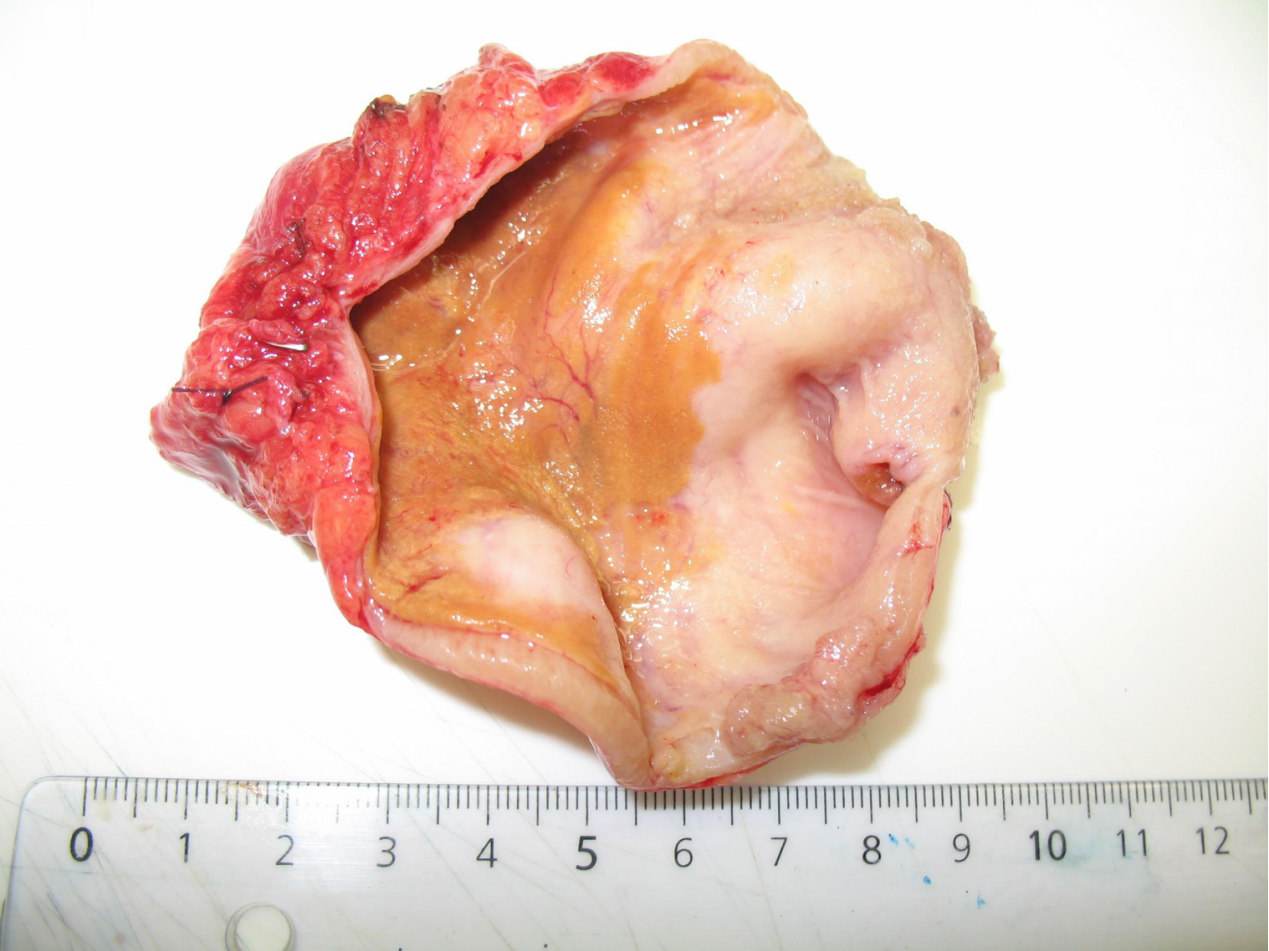
**Macroscopic picture of the opened specimen**. The luminal surface shows partly brownish deposits, the surface is irregular but smooth. The thickness of the walls is uniformly 3 mm.

During explorative laparotomy a cystic mass was found in the mesenterium which looked similar to the small bowel but had no connection to the alimentary tract (Figure 2). The surface was smooth and without deposits. An en-bloc resection of the cystic mass could be performed without the necessity of a small bowel resection. At the end of the operation the specimen was opened (Figure 3). It contained an odorless, cloudy liquid. The luminal surface showed partly brownish deposits, the surface was irregular but smooth and the walls were uniformly 3 mm thick.

**Figure 4:**
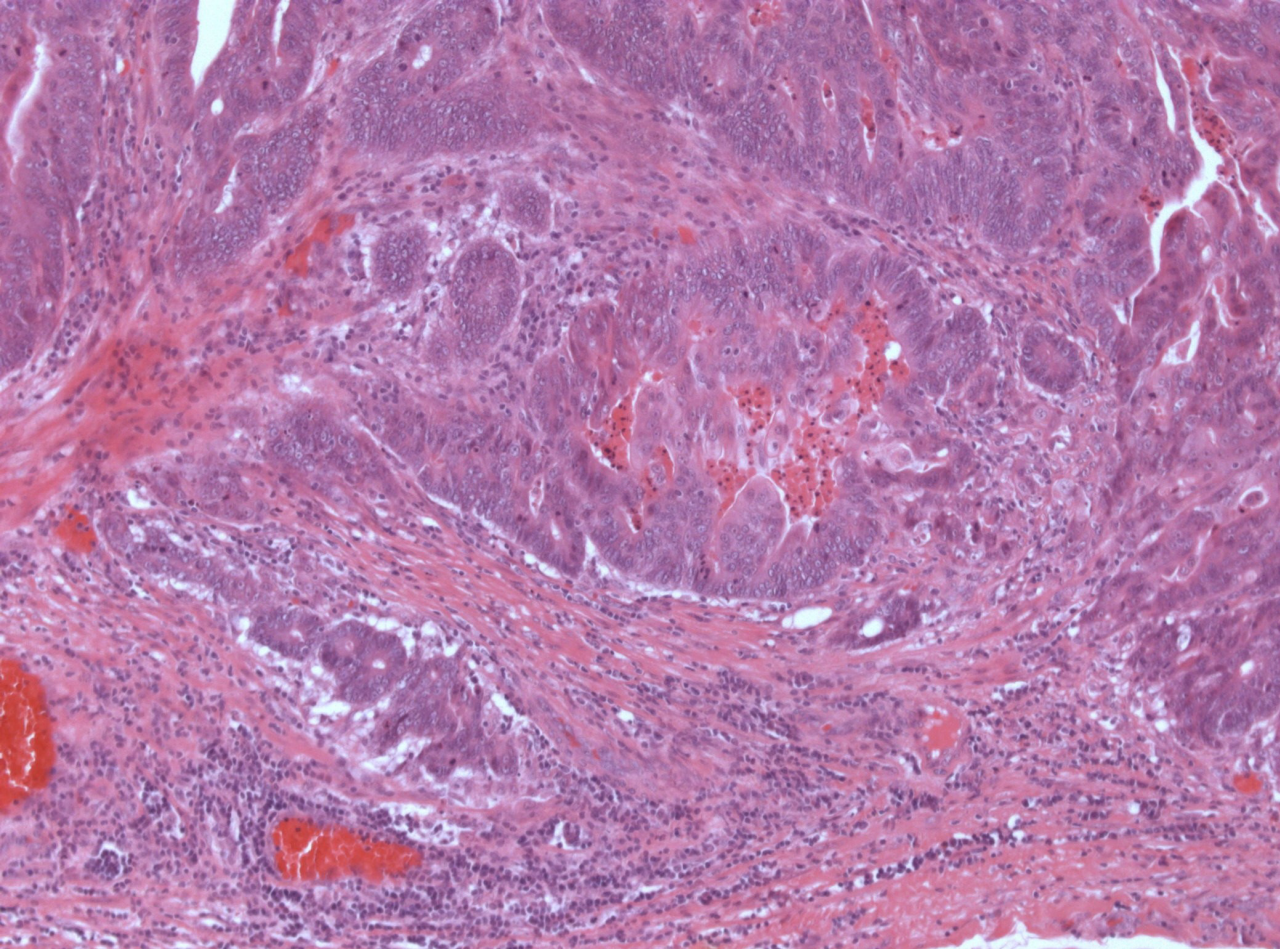
**Microscopic picture (hematoxylin and eosin stain) showing a poorly differentiated invasive adenocarcinoma (intestinal type) infiltrating the lamina muscularis propria of the enteric duplication**.

Histological examination revealed a duplication of the small bowel in the mesenterium with nearby physiological architecture. The inner lining mucosa showed indicated villi and crypts and numerous mucous cells. The epithelium showed partly dysplastic areas. At one point it contained a high-grade intraepithelial neoplasia with transition into a poorly differentiated invasive adenocarcinoma infiltrating the muscularis propria (Figure 4).

Immunohistochemistry revealed a high expression of CK20 and spot-like CK7. Analogue to the TNM-classification of the small bowel, the tumor was classified as pT2, pN0 (0/25), L0, V0, G2.

Postoperative recovery was unremarkable and the patient was discharged six days after surgery. Regular oncologic follow-up in an outpatient setting for one year after surgery showed no hints of tumor recurrence with inconspicuous physical examination and normal tumor marker levels, ultrasound, and CT findings.

## Discussion

Enteric duplications (EDs) are rare but can occur anywhere along the digestive tract from the oral cavity to the rectum [2-7,10]. The majority of ED occurs intra-abdominally and over half of them are ileal duplications [4-7]. According to W.E. Ladd, those congenital malformations involve the mesenteric side of the associated alimentary tract and share a common blood supply with the native bowel [1]. The etiology of ED still remains unknown. Several theories have been postulated such as an abnormal recanalization after the solid epithelial stage of embryonic bowel development [11]. Other theories consider persisting embryologic diverticula or 'aborted Gemini' [12]. The most accepted theory, however, is the 'intrauterine vascular accident theory' [13,14], but no single theory can explain all the known duplications [7].

EDs usually become symptomatic within the first year of life [2-7]. Reports of ED in adulthood are extremely scarce in English language literature [8]. Most frequently, the patients present vague symptoms mimicking other more common pathologies such as volvulus, appendicitis, intussusception, pelvic abscess, diverticulitis, achalasia, and Hirschsprung's disease [4,6,7,15-17].

EDs are most commonly diagnosed when complications like bowel obstruction, perforation, or bleeding occur. Prior to surgery it is difficult to diagnose EDs because of the non-specificity of symptoms and presentation. However, ultrasound, CT scan, and magnetic resonance imaging (MRI) have been useful. Ultrasound can depict the characteristic location adjacent to the bowel and the two-layered wall of EDs [15,18-20]. Bowel duplication cysts present with heterogeneous signal intensity on T1- and homogeneous signal intensity on T2-weighted images on MRI [21,22]. Latter modalities can even assist in prenatal diagnosis [22]. Where duplication is tubular, barium examination may be diagnostic if not contraindicated [7]. Technetium scanning can also be used to diagnose EDs [3]. The majority of EDs are isolated and cystic in structure. Reports of tubular duplications are less common. However, both could be associated with other malformations like intestinal malrotation and genitourinary or spinal malformations [23-25].

Heterotopic mucosa of gastric or pancreatic origin is a common finding in histological examination of ED [7]. In the current case the specimen had a similar physiological architecture to the small bowel with indicated villi, crypts, and a two-layered muscular wall. The epithelium contained many mucous cells. Gastric or pancreatic origin was not confirmed.

Carcinomas arising in duplication cysts are extremely rare complications and only few cases have been reported in literature including carcinoid tumors, squamous cell carcinomas, and common adenocarcinomas [26-50].

Malignant change in small bowel duplications is described most frequently [14,20,22,26-35], followed by colonic [36-43] and rectal [44-46] duplications. There are also reports about carcinomas arising in duplications of the duodenum [47,48] and the stomach [49,50]. Due to the rare presentation with unspecific symptoms the tumors are commonly diagnosed at advanced tumor stage with metastatic disease [26-28,35]. If malignant change is found in small bowel duplications, the high rate of lymph node metastases should be considered [26]. The mode of metastasis is similar to that of primary small bowel cancer [26,27]. Curative resections could hardly be performed [26,28]. Thus, the prognosis is generally poor once malignant change has occurred. Fortunately the suspicion of ED was a coincidental finding in an abdominal CT scan in the present case. This led to a timely operative exploration and malignant change was diagnosed at an early stage. A curative en-bloc resection of the duplication including the tumor could be performed and all of the resected 25 lymph nodes were free of metastasis.

Histological examination depicted dysplastic areas in the epithelium with an area of a high-grade intraepithelial neoplasia and transition into a poorly differentiated invasive adenocarcinoma. This indicates a tendency to undergo malignant change, which was also reported by Orr and Edwards [9]. Moreover, all cases of malignant change in duplication cysts that have been reported have occurred in adults aged 26 to 88 years. This is in contrast to the presentation of benign cysts that are diagnosed in childhood [2,4-7].

## Conclusion

The experience of this case and other reports about malignant transformation shows that whenever intestinal duplication is suspected, an immediate operative resection should be performed.

## Consent

Written consent was obtained from the patient for the use and publication of this case report and the accompanying images. A copy of the written consent is available for review from the Editor-in-Chief of this journal.

## Competing interests

The authors declare that they have no competing interests.

## Authors' contributions

GB collected the information, researched the literature, and wrote the article. AK helped with literature research and in preparing the manuscript. BS performed the histological examination and helped prepare the manuscript. RL helped in literature research and edited the final version of the manuscript. All authors read and approved the final version of the manuscript.
